# Supplementary material for: Intracranial Solitary Fibrous Tumour Management: A French Multicentre Retrospective Study
Source: Cancers (Basel). 2023 Jan 24;15(3):704. doi: 10.3390/cancers15030704 (PMC9913492; doi:10.3390/cancers15030704)
Supplement: Supplementary file 1 [file cancers-15-00704-s001.zip › cancers-2104917-supplementary.pdf]

Table S1. Initial symptoms in patients with newly diagnosed iSFT.

Initial symptoms (N=88)	n (%)
**Time between symptoms and diagnosis:**	
<1 month	29 (33%)
1-3 months	14 (16%)
3-6 months	16 (18%)
>6 months	21 (24%)
Not available	8 (9%)
**Signs of raised intracranial pressure**	
Yes	23 (26%)
No	62 (70%)
Not available	3 (3%)
**Motor deficit**	
Yes	19 (22%)
No	66 (75%)
Not available	3 (3%)
**Sensory deficit**	
Yes	8 (9%)
No	77 (88%)
Not available	3 (3%)
**Epileptic seizures**	
Yes	14 (16%)
No	71 (81%)
Not available	3 (3%)
**Cognitive disorder**	
Yes	37 (42%)
No	48 (55%)
Not available	3 (3%)
**Visual disorder**	
Yes	24 (27%)
No	61 (69%)
Not available	3 (3%)
**Cerebellar syndrome**	
Yes	14 (16%)
No	72 (82%)
Not available	2 (2%)
**Headache**	
Yes	41 (47%)
No	44 (50%)
Not available	3 (3%)

Table S2. Clinical characteristics and medical strategies in patients with distant iSFT.

Characteristics (N=16)	n (%)
**Lung metastasis**	6 (38%)
**Bone metastasis**	9 (56%)
**Liver metastasis**	5 (31%)
**Other metastasis**	6 (38%)
**Number of metastasis sites**	
1 site	10 (63%)
2 sites	3 (19%)
3 sites	2 (13%)
4 sites	1 (6%)
**Type of systemic treatment**	
Chemotherapy	5 (31%)
Targeted therapy	2 (13%)
Chemotherapy + targeted therapy	1 (6%)
None	5 (31%)
Not available	3 (19%)
**Metastasis surgery**	
Yes	8 (50%)
No	7 (44%)
Not available	1 (6%)
**Radiotherapy of metastasis**	
Yes	4 (25%)
No	11 (69%)
Not available	1 (6%)
**Best response with metastatic treatment**	
Complete response	5 (31%)
Partial response	0 (0%)
Stable disease	6 (38%)
Progression disease	3 (19%)
Not available	2 (13%)
